# Supplementary material for: HIVprotI: an integrated web based platform for prediction and design of HIV proteins inhibitors
Source: J Cheminform. 2018 Mar 9;10:12. doi: 10.1186/s13321-018-0266-y (PMC5845081; doi:10.1186/s13321-018-0266-y)
Supplement: Supplementary file 2 — Additional file 2. Source code of HIVProtI web server. [file 13321_2018_266_MOESM2_ESM.zip › HIVprotI_Source-code/hivprotI/jsmol/mathjax.htm]

JSmol-MathJax Compatibility Test


|  |  |
| --- | --- |
| When $a \ne 0$, there are two solutions to $ax^2 + bx + c = 0$, and they are $$x = {-b \pm \sqrt{b^2-4ac} \over 2a}.$$     ---    The trick here is to start the Jmol loading process only after the MathJax process is complete. We monitor the contents of the **MathJax\_Message** div every 10 ms, and while it doesn't exist or its message contains the word *Loading*, we loop:  ``` checkMathJax = function(fReady) {   return (!$("#MathJax_Message")[0]      \|\| $('#MathJax_Message:contains("Loading")')[0]     ? setTimeout(function(){checkMathJax(fReady)},10)     : fReady()); } ``` | spin on spin off |
